# Serum Trimethylamine N-Oxide Levels Correlate with Metabolic Syndrome in Coronary Artery Disease Patients

**DOI:** 10.3390/ijerph19148710

**Published:** 2022-07-18

**Authors:** Chiu-Huang Kuo, Chin-Hung Liu, Ji-Hung Wang, Bang-Gee Hsu

**Affiliations:** 1 Division of Nephrology, Hualien Tzu Chi Hospital, Buddhist Tzu Chi Medical Foundation, Hualien 97004, Taiwan; hermit.kuo@gmail.com; 2 School of Post-Baccalaureate Chinese Medicine, Tzu Chi University, Hualien 97004, Taiwan; 3 Ph.D. Program in Pharmacology and Toxicology, Department of Medicine, School of Medicine, Tzu Chi University, Hualien 97004, Taiwan; chinhung@mail.tcu.edu.tw; 4 Department of Pharmacology, Tzu Chi University, Hualien 97004, Taiwan; 5 Division of Cardiology, Hualien Tzu Chi Hospital, Buddhist Tzu Chi Medical Foundation, Hualien 97004, Taiwan; 6 School of Medicine, Tzu Chi University, Hualien 97004, Taiwan

**Keywords:** metabolic syndrome, trimethylamine N-oxide, coronary artery disease, C-reactive protein

## Abstract

Trimethylamine N-oxide (TMAO) is a gut microbial metabolite that affects atherogenesis and glucose dysregulation. The purpose of this study was to look at the link between blood TMAO levels and metabolic syndrome (MetS) in individuals with coronary artery disease (CAD). Blood samples were obtained in fasting status, and serum TMAO level was quantified by high-performance liquid chromatography–mass spectrometry. MetS and its components were defined according to the International Diabetes Federation diagnostic criteria. Of 92 enrolled patients, 51 (55.4%) had MetS. Patients with MetS had a greater proportion of hypertension and diabetes mellitus, higher body weight, waist circumference, body mass index, systolic blood pressure, fasting glucose, triglycerides, blood urea nitrogen, creatinine, C-reactive protein (CRP), insulin level, homeostasis model assessment of insulin resistance, and TMAO level. Multivariable logistic regression models revealed that TMAO level (odds ratio: 1.036, 95% confidence interval: 1.005–1.067, *p* = 0.023) could be an effective predictor of MetS among the CAD population. In these patients, the log-TMAO level was positively associated with log-CRP (β = 0.274, *p* = 0.001) and negatively associated with eGFR (β = −0.235, *p* = 0.022). In conclusion, our study revealed a positive association between serum TMAO level and MetS among patients with CAD.

## 1. Introduction

Metabolic syndrome (MetS), which is a constellation of insulin resistance, hyperglycemia, hyperlipidemia, and hypertension, was reported to have a worldwide prevalence of 10–40% and to predispose to type 2 diabetes and cardiovascular disease [1]. MetS is associated with increased cardiovascular outcomes and all-cause mortality [2]. Patients with coronary artery disease (CAD) concomitant with MetS have an increased risk of cardiovascular morbidity after follow-up [1].

Trimethylamine N-oxide (TMAO) is a metabolite that is derived from gut microbiota, comprises choline and L-carnitine, and is converted from trimethylamine by the liver enzyme flavin monooxygenase 3 (FMO3) [3]. Multiple studies have demonstrated that TMAO is an evident predictor of cardiovascular disease prevalence and the increased incidence of major adverse cardiovascular events, such as myocardial infarction, stroke, and cardiovascular mortality [4], especially in patients with preexisting CAD [5]. Recent evidence in mice revealed that TMAO could induce M1 macrophage polarization and cause pro-inflammatory environment and platelet aggregation, while reducing TMAO level stabilized atherosclerotic plaque via macrophage M2 polarization [6,7]. In addition, TMAO has been linked to obstruct the hepatic insulin signaling pathway, and the correlations between TMAO and diabetes risk appeared to be more reliable than those for cardiovascular risk [8]. Accordingly, recent studies proposed that TMAO could be a novel biomarker for MetS [9]. The purpose of this study was to look into the link between blood TMAO levels and MetS in individuals with CAD.

## 2. Materials and Methods

### 2.1. Patients

**Figure 1:**
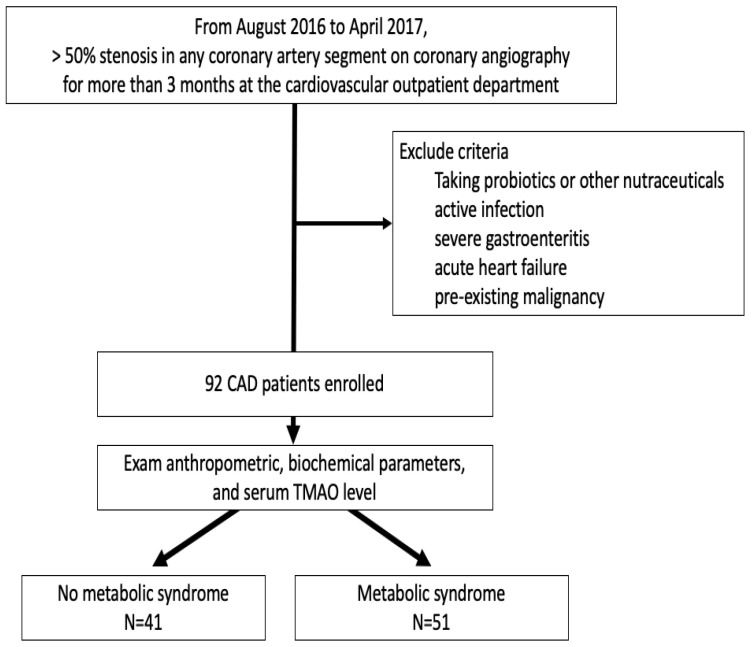
The study flow chart.

From August 2016 to April 2017, 92 patients who were proven to have greater than 50% stenosis in any coronary artery segment on coronary angiography for more than three months at the cardiovascular outpatient department at Hualien Tzu Chi Hospital were included in this study. Coronary revascularization was performed according to 2021 American College of Cardiology/American Heart Association/Society for Cardiovascular Angiography and Interventions (ACC/AHA/SCAI) guidelines. The dual antiplatelet agents were oral daily with clopidogrel 75 mg and aspirin 100 mg for at least three months after coronary revascularization, and 70 patients had coronary artery stents (20 patients with bare-metal stents and 50 patients with drug-eluting stents). This study protocol was permitted by the Hualien Tzu Chi Hospital Research Ethics Committee (IRB108-96-B). Active infection, severe gastroenteritis, heart failure at the time of blood sampling, consuming probiotics or other nutraceuticals, or a pre-existing malignancy were all reasons for patients to be excluded from the study. Hypertension and diabetes were classified according to the ICD10 diagnosis in medical records, or via a prescription for antihypertensive/antidiabetic agents. Figure 1 depicts the flow chart of this study.

### 2.2. Anthropometric Analysis

Bodyweight, body height, and waist circumference were measured simultaneously. The body mass index (BMI) was calculated by dividing the subjects’ measured weight (kg) by their height squared (m^2^).

### 2.3. Biochemical Investigations

Venous blood was collected after an overnight fasting period. We measured blood urea nitrogen (BUN), creatinine, fasting glucose, low-density lipoprotein cholesterol (LDL-C), high-density lipoprotein cholesterol (HDL-C), total cholesterol (TCH), triglycerides (TG), and C-reactive protein (CRP). An enzyme-linked immunosorbent test (ELISA) was used to measure serum insulin levels. (Labor Diagnostika Nord, Nordhorn, Germany). The homeostasis model assessment-estimated IR (HOMA-IR) was used to estimate insulin sensitivity according to the following formula: fasting plasma insulin (μU/mL) × fasting plasma glucose (mg/dL)/405 [10]. The estimated glomerular filtration rate (eGFR) was calculated using the Chronic Kidney Disease Epidemiology Collaboration equation.

### 2.4. Metabolic Syndrome and Its Components

The definition of MetS required more than three of the following five components according to International Diabetes Federation diagnostic criteria [11]: (1) central obesity (waist circumference ≥ 90 cm for men or ≥80 cm for women); (2) systolic blood pressure ≥ 130 mmHg or diastolic ≥ 85 mmHg; (3) fasting glucose ≥ 100 mg/dL; (4) HDL-cholesterol < 50 mg/dL for women and <40 mg/dL for men); (5) TG ≥ 150 mg/dL.

### 2.5. High-Performance Liquid Chromatography-Mass Spectrometry

The serum TMAO levels were determined using a Waters e2695 high-performance liquid chromatography system with a mass spectrometer (ACQUITY QDa, Waters Corporation, Milford, MA, USA) [12]. To monitor the participants’ compound (TMAO: 76.0 *m*/*z*; d9-TMAO: 85.1 *m*/*z*), mass spectrometry was used with complete scan ranges of 50–450 *m*/*z* for positive-ion modes and 100–350 *m*/*z* for negative-ion modes. TMAO and d9-TMAO had a retention time of 2.54 min. The Empower^®^ 3.0 program was used to collect and analyze all of the examinations (New York, NY, USA).

### 2.6. Statistical Analysis

The Kolmogorov–Smirnov test was used to determine whether continuous variables had a normal distribution. The Mann–Whitney U test was used to compare nonnormally distributed data such as TG, fasting glucose, BUN, creatinine, CRP, insulin, HOMA-IR, and TMAO. Data expressed as the number of patients was analyzed by the χ2 test. Variables significantly associated with MetS were tested for independence by multivariate logistic regression analysis. Because serum insulin and HOMA-IR levels had multicollinearity with variance inflation factor > 10 with MetS. We did not add insulin and HOMA-IR in multivariate logistic regression analysis for MetS. The nonnormally distributed variables underwent logarithmic transformations with base 10 to achieve normality. In a simple linear regression analysis, variables significantly linked with logarithmically transformed TMAO (log-TMAO) were checked for independence, followed by a multivariate forward stepwise regression analysis. The efficiency of the prediction models was assessed using the areas under the receiver operating characteristic (ROC) curve generated by the logistic regression model. Data were analyzed using SPSS for Windows (version 19.0; SPSS Inc., Chicago, IL, USA). Values of *p  *< 0.05 were considered to be statistically significant.

## 3. Results

The demographic, biochemical, and clinical characteristics of the 92 CAD patients included in this study are shown in Table 1; of these, 51 patients (55.4%) had MetS. Compared with patients without MetS, those with MetS had significantly higher percentages of hypertension (*p* < 0.001) and DM (*p* < 0.001); significantly higher body weight (*p* = 0.004), waist circumference (*p* < 0.001), BMI (*p* < 0.001), SBP (*p* = 0.002), fasting glucose (*p* = 0.003), TG (*p* < 0.001), BUN (*p* = 0.003), creatinine (*p* = 0.004), CRP (*p* = 0.048), insulin level (*p* = 0.041), HOMA-IR (*p* = 0.004), and TMAO level (*p* < 0.001); and significantly lower HDL-C (*p* = 0.006) and eGFR (*p* < 0.001).

**Figure 2:**
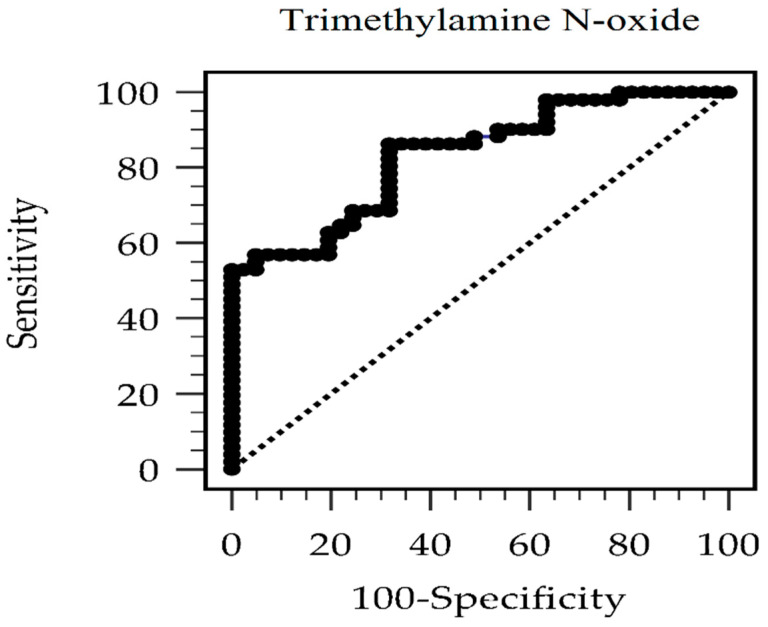
ROC curve for metabolic syndrome prediction by trimethylamine N-oxide level.

Table 2 shows the odds ratio (OR) of TMAO for MetS after multivariate logistic regression analysis. The unadjusted serum TMAO levels with MetS revealed that for every 1 μg/L that TMAO increased, the risk of MetS increased by 3.4% [OR: 1.034, 95% confidence interval (CI): 1.017–1.052, *p* < 0.001]. Model 1 was adjusted for the MetS components, such as waist circumference, DM, hypertension, fasting glucose, TG, and HDL-C. Model 1 showed a 3.3% increase in the risk of MetS (OR: 1.033, 95% CI: 1.009–1.058, *p* = 0.007) for every 1-μg/L increase in TMAO level. In addition to the variables in model 1, other variables that were significant for MetS (i.e., BMI, eGFR, CRP, insulin level, and HOMA-IR) were included in model 2. Model 2 showed a 4.3% increase in the risk of MetS (OR: 1.043, 95% CI: 1.001–1.087, *p* = 0.043) for every 1-μg/L increase in TMAO level. The result above suggested that TMAO had a positive association with MetS in patients with CAD after adjusting for significant confounders. According to the ROC curve, the optimal cutoff serum value of TMAO for predicting MetS in patients with CAD was 106.69 g/L, with an area under the ROC curve of 0.832 (95 percent CI 0.739–0.902, p 0.001), a sensitivity of 86.3 percent, and a specificity of 68.3 percent (Figure 2).

Simple multivariate linear analyses positively correlated log-TMAO level with hypertension (*r* = 0.260, *p* = 0.012); waist circumference (*r* = 0.279, *p* = 0.007); SBP (*r* = 0.265, *p* = 0.011); log-BUN (*r* = 0.246, *p* = 0.018); log-creatinine (*r* = 0.214, *p* = 0.041); log-CRP (*r* = 0.335, *p* = 0.001) and was negatively correlated with eGFR (*r* = −0.306, *p* = 0.003) (Table 3). In a multivariate forward stepwise linear regression model, log-CRP (β = 0.274, adjusted R^2^ change = 0.103, *p* = 0.001) and eGFR (β = −0.235, adjusted R^2^ change = 0.042, *p* = 0.022) were independently and significantly associated with log-TMAO levels.

## 4. Discussion

This study on patients with CAD found that the fasting TMAO level was positively associated with MetS. In addition, log-TMAO level was positively associated with log-CRP level and negatively associated with eGFR.

Beyond the connection between atherosclerosis and poor cardiovascular outcomes, increasing evidence suggested that gut microbiota is crucial in glucose hemostasis [13]. A recent meta-analysis suggested that high levels of serum TMAO were associated with an increased risk of DM [14]. The precise mechanism of the effects of TMAO on insulin resistance remains unclear. The proposed pathway was the TMAO-dependent elevation of N-nitroso compounds, which induce DM [4]. Dietary TMAO was shown to impair hepatic insulin transduction, deteriorate glucose tolerance, and cause adipose tissue inflammation in mice fed with a high-fat diet [15]. Furthermore, high levels of plasma TMAO could decrease the synthesis and transport the proteins of bile acids, which could regulate glucose metabolism through several pathways [16]. Finally, the inhibition of FMO3 reduced TMAO levels and lowered serum glucose in murine [17].

Several reports revealed that TMAO could alter lipid homeostasis. First, TMAO was shown to promote foam cell formation by upregulating macrophage scavenger receptors [18]. Second, TMAO inhibited hepatic bile acid synthesis by the downregulation of Cyp7a1 expression, which is the rate-limiting step in cholesterol catabolism [19]. As a critical enzyme of TMAO, FMO3 may promote hepatic lipogenesis and gluconeogenesis and impair transintestinal cholesterol export [16]. Moreover, direct supplementation of TMAO could enhance atherosclerotic lesion development in mice [20].

Regarding the last two components of MetS, there is limited literature on the correlation of TMAO with hypertension and obesity. In one rat study, TMAO prolonged the angiotensin II ability to elevate blood pressure, which is the crucial component of the renin-angiotensin system [21]. In one human study with obese subjects, the TMAO level was associated with visceral fat mass and liver fat content [22]. The pharmacologic inhibition of FMO3 reportedly stimulated white adipose tissue to turn into beige adipose tissue, which meant that the inhibition of FMO3 promotes resistance to obesity [23]. We found that serum TMAO levels had positive correlations with waist circumference, SBP, and hypertension in patients with CAD. Antihypertensive agents including angiotensin-converting enzyme inhibitor, angiotensin-receptor blocker, β-blocker, or calcium-channel blocker or the statin or fibrate used revealed no significant correlation to log-TMAO levels in this study.

Several confounders could alter serum TMAO levels, and one of the most important is renal function. TMAO is cleared by the kidney and excreted unchanged through the urine. Nearly 95% of TMAO is excreted in the urine within one day [24]. Currently, the organic cation transporter 2 is the critical channel for TMAO uptake [25]. In addition, increased TMAO concentration was observed to normalize after renal transplantation [26]. A meta-analysis that included 13,783 participants noted that a circulating TMAO level was positively associated with CRP on both two-class and dose-response meta-analyses [27]. Our study revealed that serum TMAO levels were associated with renal function and CRP level in patients with CAD. The log-TMAO level was found to be negatively linked with eGFR after a multivariate linear regression analysis.

The intestinal microbiota plays a pivotal role in cardiovascular diseases and vascular aging [28,29]. With more knowledge about TMAO in cardiovascular disease and studies of trimethylaminuria (TMAU), urine TMA/TMANO ratios could be a feasible clinical marker instead of serum TMAO. Furthermore, FMO3 genotyping and intestinal microbiota analysis might be the relevant steps to determine etiopathogenesis [30,31]. Beyond TMAO, recent research suggested that nutraceuticals may play an essential role in MetS management and affect the microbiome and oxidative stress [32].

The cross-sectional design of this study, as well as the small number of patients included, and a post-hoc analysis noted the power is 0.655, were limitations of this work. Only the correlation of TMAO with MetS was provided instead of causality. There was no diet pattern evaluation or microbiome analysis of the subjects to prevent synthesis differences. In addition, the discrepancies in sex, CRP level, estrogen concentration, and inflammation severity may have confounded the TMAO levels. The highly positive correlation between serum TMAO and urine TMAO suggests that urine TMAO has the potential to serve as clinical applicability in the future [33]. Moreover, a recent study also noted that lower ratios of urine to plasma concentrations of TMAO were associated with cardiovascular and all-cause mortality in diabetic kidney disease [34]. Therefore, further studies are warranted to validate the TMAO levels for MetS in patients with CAD.

## 5. Conclusions

In conclusion, serum fasting TMAO level was positively associated with MetS, CRP level, and eGFR in patients with CAD.

## Figures and Tables

**Table 1:** Demographic and clinical characteristics of the study population.

Variables	All Patients (*n* = 92)	No Metabolic Syndrome Group (*n* = 41)	Metabolic Syndrome Group (*n* = 51)	*p* Value
Age (years)	65.44 ± 9.37	66.29 ± 8.77	64.75 ± 9.86	0.435
Height (cm)	161.30 ± 7.88	161.54 ± 6.26	161.12 ± 9.03	0.802
Body weight (kg)	68.59 ± 12.19	64.59 ± 9.51	71.82 ± 13.20	0.004 *
Waist circumference (cm)	92.52 ± 10.20	86.98 ± 7.99	96.98 ± 9.63	<0.001 *
Body mass index (kg/m^2^)	26.26 ± 3.59	24.73 ± 3.13	27.50 ± 3.48	<0.001 *
Systolic blood pressure (mmHg)	130.27 ± 16.47	124.44 ± 13.64	134.96 ± 17.16	0.002 *
Diastolic blood pressure (mmHg)	72.25 ± 10.29	70.73 ± 8.33	73.47 ± 11.56	0.206
Total cholesterol (mg/dL)	167.36 ± 37.17	164.85 ± 33.71	169.37 ± 39.94	0.565
Triglycerides (mg/dL)	120.00 (91.25–183.00)	104.00 (86.50–127.50)	151.00 (101.00–238.00)	<0.001 *
HDL-C (mg/dL)	45.41 ± 12.15	49.27 ± 13.49	42.31 ± 10.05	0.006 *
LDL-C (mg/dL)	96.23 ± 27.47	95.56 ± 27.1	96.76 ± 28.00	0.836
Fasting glucose (mg/dL)	113.00 (98.25–157.00)	100.00 (92.00–146.50)	125.00 (105.00–157.00)	0.003 *
Blood urea nitrogen (mg/dL)	16.00 (13.00–20.00)	15.00 (12.00–17.50)	19.00 (13.00–22.00)	0.003 *
Creatinine (mg/dL)	1.10 (0.90–1.30)	1.00 (0.90–1.20)	1.20 (0.90–1.50)	0.004 *
eGFR (mL/min)	67.02 ± 19.42	75.03 ± 13.44	60.57 ± 21.14	<0.001 *
C-reactive protein (mg/dL)	0.19 (0.14–0.26)	0.18 (0.14–0.22)	0.22 (0.15–0.30)	0.048 *
Insulin (uIU/mL)	12.57 (9.34–17.13)	11.18 (7.09–15.69)	14.64 (9.89–19.60)	0.041 *
HOMA-IR	3.97 (2.81–5.39)	3.56 (2.17–4.72)	4.23 (3.30–6.22)	0.004 *
TMAO (μg/L)	119.58 (98.00–176.72)	99.96 (88.95–128.41)	153.67 (109.41–219.80)	<0.001 *
Female (*n*, %)	21 (22.8)	6 (14.6)	15 (29.4)	0.093
Hypertension (*n*, %)	72 (78.3)	24 (58.5)	48 (94.1)	<0.001 *
Diabetes (*n*, %)	41 (44.6)	10 (24.4)	31 (60.8)	<0.001 *
ACE inhibitor use (*n*, %)	22 (23.9)	6 (14.6)	16 (31.4)	0.061
ARB use (*n*, %)	34 (37.0)	12 (29.3)	22 (43.1)	0.171
β-blocker use (*n*, %)	52 (56.5)	21 (51.2)	31 (60.8)	0.358
CCB use (*n*, %)	34 (37.0)	11 (26.8)	23 (45.1)	0.071
Statin use (*n*, %)	64 (69.6)	25 (61.0)	39 (76.5)	0.108
Fibrate use (*n*, %)	15 (16.3)	4 (9.8)	11 (21.6)	0.127

The categorial variables are presented as count and percentage; the continuous values are represented as median (interquartile range) or mean ± standard deviation. Abbreviations: LDL-cholesterol, low density lipoprotein cholesterol; HDL-cholesterol, high-density lipoprotein cholesterol; eGFR, estimated glomerular filtration rate; HOMA-IR, homeostasis model assessment of insulin resistance; TMAO, Trimethylamine N-oxide; ACE, angiotensin-converting enzyme; ARB, angiotensin-receptor blocker; CCB, calcium-channel blocker. * *p* value refers to the comparison between the metabolic syndrome group and the non-metabolic syndrome group.

**Table 2:** Multivariable logistic regression investigation of serum trimethylamine N-oxide levels among 92 coronary artery disease patients.

TMAO (μg/L)	Unadjusted		Model 1		Model 2	
	OR (95% CI)	*p* Value	OR (95% CI)	*p* Value	OR (95% CI)	*p* Value
Per 1 μg/L TMAO increase	1.034 (1.017–1.052)	<0.001 *	1.033 (1.009–1.058)	0.007 *	1.036 (1.005–1.067)	0.023 *

Model 1 is adjusted for waist circumference, diabetes mellitus, hypertension, fasting glucose, triglycerides, and high-density lipoprotein cholesterol. Model 2 is adjusted for the Model 1 variables and for body mass index, estimated glomerular filtration rate and C-reactive protein. TMAO, Trimethylamine N-oxide; OR, odds ratio; CI, confidence interval. * *p* < 0.05 was considered statistically significant.

**Table 3:** Correction between log-transformed trimethylamine N-oxide level and clinical variables.

Variables	Log-Transformed TMAO (μg/L)				
	Simple Regression		Multivariate Regression		
	*r*	*p* Value	Beta	Adjusted R^2^ Change	*p* Value
Female	0.162	0.122	-	-	-
Hypertension	0.260	0.012 *	-	-	-
Diabetes	0.075	0.477	-	-	-
ACE inhibitor use	0.031	0.768	-	-	-
ARB use	0.044	0.680	-	-	-
β-blocker use	0.157	0.134	-	-	-
CCB use	0.077	0.467	-	-	-
Statin use	0.102	0.333	-	-	-
Fibrate use	0.137	0.194	-	-	-
Age (years)	0.067	0.528	-	-	-
Body weight (kg)	0.094	0.372	-	-	-
Waist circumference (cm)	0.279	0.007 *	-	-	-
Body mass index (kg/m^2^)	0.190	0.069	-	-	-
Systolic blood pressure (mmHg)	0.265	0.011 *	-	-	-
Diastolic blood pressure (mmHg)	−0.008	0.942	-	-	-
Total cholesterol (mg/dL)	0.041	0.698	-	-	-
Log-Triglyceride (mg/dL)	0.187	0.074	-	-	-
HDL-C (mg/dL)	−0.174	0.096	-	-	-
LDL-C (mg/dL)	0.008	0.941	-	-	-
Log-Glucose (mg/dL)	0.007	0.951	-	-	-
Log-BUN (mg/dL)	0.246	0.018 *	-	-	-
Log-Creatinine (mg/dL)	0.214	0.041 *	-	-	-
eGFR (mL/min)	−0.306	0.003 *	−0.235	0.042	0.022 *
Log-CRP (mg/dL)	0.335	0.001 *	0.274	0.103	0.001 *
Log-Insulin (uIU/mL)	0.125	0.235	-	-	-
Log-HOMA-IR	0.114	0.279	-	-	-

Data of triglycerides, glucose, BUN, creatinine, CRP, insulin, HOMA-IR, and TMAO values were log-transformed before analysis. Simple linear regression or multivariate stepwise linear regression analysis performed with adopted factors (hypertension, waist circumference, systolic blood pressure, log-BUN, log-creatinine, eGFR and log-CRP). ACE, angiotensin-converting enzyme; ARB, angiotensin-receptor blocker; CCB, calcium-channel blocker; HDL-C, high density lipoprotein cholesterol; LDL-C, low density lipoprotein cholesterol; BUN, blood urea nitrogen; eGFR, estimated glomerular filtration rate; CRP, C-reactive protein; HOMA-IR, homeostasis model assessment of insulin resistance; TMAO, trimethylamine N-oxide. * Statistical significance was regarded as *p* < 0.05.

## Data Availability

The data presented in this study are available on request from the corresponding author.
